# Translating CLEOPATRA into routine practice: National treatment patterns and survival for patients with HER2-positive metastatic breast cancer

**DOI:** 10.1016/j.breast.2026.104852

**Published:** 2026-06-27

**Authors:** Kathrine F. Vandraas, Sarah Hjorth, Cassia B. Trewin-Nybråten, Bettina K. Andreassen, Egil S. Blix, Bjørn Naume, Edoardo Botteri, Giske Ursin, Nathalie C. Støer

**Affiliations:** a Department of Research, Cancer Registry of Norway, Norwegian Institute of Public Health, Oslo, Norway; b Norwegian Research Center for Women's Health, Department of Obstetrics and Gynecology, Oslo University Hospital, Oslo, Norway; c Department of Oncology Oslo University Hospital, Oslo, Norway; d Department of Registration, Cancer Registry of Norway, Norwegian Institute of Public Health, Oslo, Norway; e Department of Clinical Medicine, The Arctic University of Norway, Tromsø, Norway; f Department of Oncology, University Hospital of North Norway, Tromsø, Norway; g Institute of Clinical Medicine, University of Oslo, Oslo, Norway; h Department of Colorectal Cancer Screening, Cancer Registry of Norway, Norwegian Institute of Public Health, Oslo, Norway; i Cancer Registry of Norway, Norwegian Institute of Public Health, Oslo, Norway; j Institute of Basic Medical Sciences, University of Oslo, Oslo, Norway; k Department of Preventive Medicine, University of Southern California, Los Angeles, CA, USA

**Keywords:** HER2-Positive breast cancer, Real-world data, CLEOPATRA

## Abstract

**Introduction:**

Amplification of the epidermal growth factor receptor 2 (HER2) gene occurs in 15-20% of breast cancers and was historically associated with poor prognosis. The CLEOPATRA trial established pertuzumab added to trastuzumab and chemotherapy as standard of care for metastatic HER2-positive breast cancer, demonstrating a 15.7-month improvement in median overall survival (OS). Strict trial inclusion criteria may limit generalizability to clinical practice. Population-based data can assess whether these survival benefits translate to real-world outcomes.

**Methods:**

Women diagnosed with HER2-positive metastatic breast cancer in 2012–2021 were identified through the Cancer Registry of Norway and linked to health and treatment databases. Patients were categorized by treatment regimen and year of treatment initiation: pre-CLEOPATRA (2012–2014; trastuzumab ± chemotherapy) and post-CLEOPATRA (2015–2021; trastuzumab + pertuzumab ± chemotherapy). OS was estimated using Kaplan–Meier and Cox regression adjusted for clinical, and socioeconomic factors. Outcomes were benchmarked against CLEOPATRA.

**Results:**

The cohort comprised 329 women, 23% were aged ≥65 years. Seventy-eight patients (24%) received pre-CLEOPATRA regimens and 251 (76%) post-CLEOPATRA regimens; 59% met minimum one CLEOPATRA exclusion criterion. First-line dual HER2 blockade increased from 0% in 2012 to 75% in 2021. Median OS improved from 69 months to 96 months post-CLEOPATRA. Four-year OS increased from 59% to 65% (adjusted hazard ratio 0.77, 95% CI 0.53–1.12). Real-world OS was consistent with CLEOPATRA outcomes.

**Conclusions:**

Nationwide real-world data demonstrate favorable survival outcomes following adoption of dual anti-HER2 therapy. While these findings are broadly consistent with the CLEOPATRA trial, the observational design does not allow direct assessment of equivalence between studies.

**Abbreviations:**

all abbreviations in the abstract are standard in this field of research.

## Highlights

• Real-world survival of dual anti-HER2 therapy appears consistent with outcomes reported in the pivotal CLEOPATRA-trial.
• This nation-wide cohort supports the effectiveness of dual anti-HER2 blockade in clinical practice.
• Population-based health registries complement knowledge derived from pivotal clinical trials.

## Graphical abstract

**Figure:**
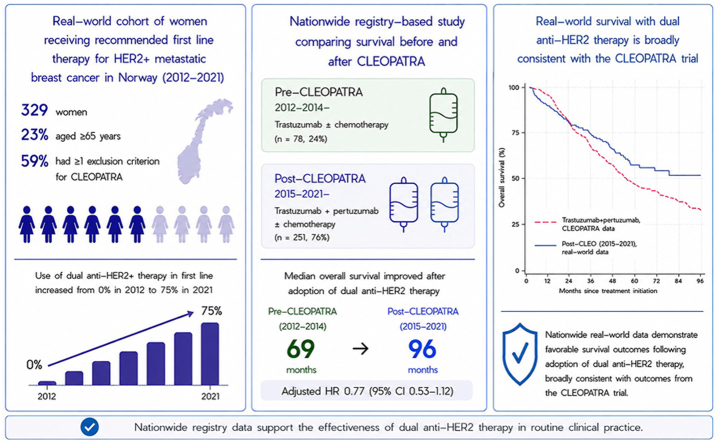

## Glossary:

HER2: human epidermal growth factor receptor 2
OS: overall survival
BC: breast cancer
mBC: metastatic breast cancer
CRN: Cancer Registry of Norway
NPR: National Patient Registry
NorPD: Norwegian Prescription Database
ICD: international classification of diseases
SACT-database: systemic anti-cancer treatment database

## 1 Introduction

HER2 amplification occurs in about 15-20% of breast cancers (BC) and historically conferred poor prognosis in the metastatic setting (mBC) [1]. The introduction of trastuzumab transformed outcomes, and the CLEOPATRA trial later demonstrated that adding pertuzumab to trastuzumab and chemotherapy significantly improved survival compared to trastuzumab alone [2,3]. Median overall survival (OS) improved by 15.7 months (56.5 vs. 40.8 months) at four years [2], with the benefit maintained after eight years of follow-up [4]. These results were practice-changing, and dual HER2 blockade has since been established as standard first-line therapy worldwide [5,6].

CLEOPATRA enrolled a highly selected population, excluding patients with brain metastasis, comorbidities and impaired performance status. Only 3% of participants were older than 65 years [1]. Consequently, the trial population does not fully represent the heterogeneous patient population encountered in clinical practice, raising questions about the external validity of its findings. Several real-world studies have since confirmed the effectiveness of dual HER2 blockade, but data remain limited, often restricted to subsets of patients with shorter follow-up [[7], [8], [9], [10]].

Norway offers a unique setting to evaluate the generalizability of CLEOPATRA. Cancer care is universally accessible and delivered almost exclusively through public hospitals, and national health registries provide high-quality, longitudinal and individualized data on tumor characteristics, systemic therapy, comorbidity and survival. These features enable population-wide assessment of treatment patterns and outcomes with minimal loss to follow-up.

In this study, we examined national treatment patterns and long-term outcomes for HER2+ mBC patients in Norway across the period spanning the introduction of pertuzumab. Specifically, we compared survival among those treated with trastuzumab ± chemotherapy before the CLEOPATRA regimen became standard of care to survival among those treated with trastuzumab and pertuzumab ± chemotherapy after the CLEOPTATRA regime was introduced, and benchmark real-world outcomes against those reported in the pivotal trial.

## 2 Material and methods

Women aged ≥18 years registered in the Cancer Registry of Norway (CRN) with a diagnosis of HER2-positive mBC from 2012 to 2021 were identified. During this period, in 2015, the treatment guidelines changed from trastuzumab monotherapy + chemotherapy to dual anti-HER2 therapy + chemotherapy. CRN data were linked to the National Patient Registry (NPR), Statistics Norway, the Norwegian Cause of Death Registry, and the Norwegian Prescription Database (NorPD) using the unique personal identification number assigned to all Norwegian residents.

A total of 764 HER2+ patients with either de novo metastatic disease (stage IV at diagnosis or within four months of primary diagnosis) or relapse after early-stage HER2+ BC were initially identified. When multiple BC diagnoses were registered on the same day, classification was based on the most aggressive histology. Exclusion criteria were previous cancer diagnosis (other than non-melanoma skin cancer, n = 88); prior cancer therapy without a registered diagnosis (n < 5); simultaneous diagnosis of another cancer (n < 5); diagnosis at autopsy, dead or censored at the date of metastatic diagnosis (n < 5); and missing data on adjustment variables (n = 8). Additionally, patients with no registered use of trastuzumab in 2012–2014, or dual anti-HER2 therapy from 2015 and later, were excluded (n = 348). The final study sample comprised 329 women with HER2+ mBC, followed from initiation of first metastatic anti-HER2 therapy until death or June 2023, whichever occurred first (Supplementary Fig. 1).

Based on individual level data and treatment era, two cohorts were defined: the pre-CLEOPATRA cohort (2012–2014), including patients receiving trastuzumab monotherapy ± chemotherapy, and the post-CLEOPATRA cohort (2015–2021), including patients receiving trastuzumab plus pertuzumab ± chemotherapy, reflecting the 2015 approval of the combination in Norway. The study population represents patients who received recommended first line anti-HER2 therapy according to recommendations in each era and does not include all patients diagnosed with HER2-positive metastatic breast cancer in Norway during the study period.

**Clinical variables**: Tumor characteristics and dates of metastasis were obtained from the CRN, including data from the Norwegian Breast Cancer Registry [11,12]. Metastatic disease was identified using clinical and pathology reports from the CRN, supplemented with ICD-10 codes for metastatic disease (C77–C79) from NPR. Patients recorded in the CRN receiving stereotactic or whole-brain radiotherapy were categorized as having brain metastases, as these lesions are often diagnosed without biopsy. The Norwegian Cause of Death Registry provided dates and causes of death, which were used to calculate survival time from the date of first-line treatment start to death.

**Systemic cancer treatment**: Data on systemic cancer therapies administered in Norwegian public hospitals (excluding Northern Norway) were obtained from the CRN systemic anti-cancer treatment database (SACT) [13]. Coverage of systemic treatments is approximately 91% for 2019–2021, with partial data available for earlier years. Treatment information for Northern Norway and for years not covered by SACT was derived from the NPR. Outpatient therapies, including endocrine and other orally administered cancer drugs dispensed by pharmacies, were captured through the Norwegian Prescribed Drug Registry (NorPD).

**General health variables:** Patient comorbidity was assessed using ICD-10 codes from the NPR registered during the four years prior to inclusion, supplemented by drug reimbursement codes from NorPD within the year preceding inclusion. Definitions of cardiovascular disease, chronic obstructive pulmonary disease (COPD), and diabetes followed the Non-Communicable Diseases in Norway project [14], while kidney failure and dementia were defined using ICD-10 codes (N17-19, F00-03 or G30). The Patient Registry Index (PRI), a modified Charlson comorbidity index, was also calculated; values above zero indicate hospital admissions for one or more comorbid conditions (excluding cancer) [15]. Polypharmacy was defined as having eight or more different prescriptions registered in NorPD during the year prior to inclusion. During the same period, the number of hospital contacts unrelated to cancer was recorded and categorized as 0, 1–2, or ≥3.

**Sociodemographic variables:** Educational attainment was categorized as compulsory (elementary), secondary (high school), or higher (university). Household income was classified into quintiles based on the median income of the general Norwegian population in the year of diagnosis. Both variables were provided by Statistics Norway.

**CLEOPATRA exclusion criteria:** among the 23 inclusion criteria set for CLEOPATRA [2], we could define information on the following variables using ICD-10 codes from the NPR; current peripheral neuropathy, severe hypertension, unstable angina, congestive heart failure, dyspnea, severe systemic disease, infections, or receipt of intravenous antibiotics. Treatment with corticosteroids was obtained from the NorPD. Information on prior cancer therapies was obtained as described for the treatment variables. Patients who were registered with at least one of these variables were labelled in Table 1 as CLEOPATRA non-eligible.

**Table 1:** Baseline characteristics of real world study sample (n = 329), in total and according time period and treatment cohort (pre- and post CLEOPATRA).

	2012-2021	2012-2014 (Pre-CLEOPATRA)	2015-2021 (Post- CLEOPATRA
**In total (n)**	329	78	251
**Sociodemographics**			
Age, yr			
Median (IQR)	54 (45-64)	54 (43-63)	53 (46-64)
Range	21-88	24-88	21-82
<65	252 (76.6%)	62 (79.5%)	190 (75.7%)
65-74	53 (16.1%)	9 (11.5%)	44 (17.5%)
≥75	24 (7.3%)	7 (9.0%)	17 (6.8%)
**Year of first anti-HER2 treatment line**			
2012-2014	78 (23.7%)	78 (100.0%)	0
2015-2017	84 (25.5%)	0	84 (33.5%)
2018-2021	167 (50.8%)	0	167 (66.5%)
**Educational attainment**^a^			
Compulsary	64 (19.6%)	19 (24.4%)	45 (18.1%)
Secondary	141 (43.3%)	34 (43.6%)	107 (43.1%)
Higher	121 (37.1%)	25 (32.1%)	96 (38.7%)
Missing	(n < 5)	0	(n < 5)
**Household income**			
Lowest quintile	67 (20.4%)	19 (24.4%)	48 (19.1%)
Middle 3 quintiles	193 (58.7%)	44 (56.4%)	149 (59.4%)
Highest quintiles	69 (21.0%)	15 (19.2%)	54 (21.5%)
**Comorbidities**			
Cardiovascular disease	74 (22.5%)	18 (23.1%)	56 (22.3%)
Chronic obstructive pulmonary disease	7 (2.1%)	(n < 5)	5 (2.0%)
Dementia	0	0	0
Diabetes	19 (5.8%)	(n < 5)	16 (6.4%)
Kidney failure	(n < 5)	0	(n < 5)
**Patient registry index >0**	40 (12.2%)	9 (11.5%)	31 (12.4%)
**Polypharmacy**	190 (57.8%)	46 (59.0%)	144 (57.4%)
**Hospital contacts during the year prior to treatment start**			
0	129 (39.2%)	35 (44.9%)	94 (37.5%)
1-2	135 (41.0%)	29 (37.2%)	106 (42.2%)
3+	65 (19.8%)	14 (17.9%)	51 (20.3%)
**Clinical characteristics**			
Hormone receptor positive	197 (59.9%)	50 (64.1%)	147 (58.6%)
Relapsed from early-stage BC	170 (51.7%)	31 (39.7%)	139 (55.4%)
**Curative treatment**			
No	204 (62.0%)	54 (69.2%)	150 (59.8%)
Yes	125 (38.0%)	24 (30.8%)	101 (40.2%)
Anthracycline^b^	107 (85.6%)	20 (83.3%)	87 (86.1%)
Endocrine therapy^c^	63 (82.9%)	6 (37.5%)	57 (95.0%)
Taxane^b^	120 (96.0%)	21 (87.5%)	99 (98.0%)
Trastuzumab^b^	110 (88.0%)	21 (87.5%)	89 (88.1%)
**First line metastatic treatment**	329 (100.0%)	78 (100.0%)	251 (100.0%)
Trastuzumab^d^	78 (23.7%)	78 (100.0%)	0
Trastuzumab + pertuzumab^d^	251 (76.3%)	0	251 (100.0%)
**Second line metastatic treatment**^a^	151 (47.3%)	32 (45.1%)	119 (48.0%)
Trastuzumab^d^	46 (30.5%)	0	46 (38.7%)
Trastuzumab + pertuzumab^d^	19 (12.6%)	19 (59.4%)	0
Trastuzumab emtansine^d^	68 (45.0%)	6 (18.8%)	62 (52.1%)
Trastuzumab deruxtecan^d^	6 (4.0%)	(n < 5)	5 (4.2%)
Trastuzumab + lapatinib^d^	(n < 5)	(n < 5)	(n < 5)
Lapatinib^d^	8 (5.3%)	(n < 5)	(n < 5)
Unspecified^e^	10	7	(n < 5)
**CLEOPATRA ineligible**^f^	196 (59.6%)	50 (64.1%)	146 (58.2%)

Abbreviations: inter quartile range (IQR), breast cancer (BC), polypharmacy: defined as 8+ different medications in the year before first treatment cycle.

a Proportion of non-missing values.

b Proportion of curatively treated. More than one type of treatment may have been given.

c Proportion of curatively treated with hormone receptor positive disease. More than one type of treatment may have been given.

d Proportion of patients who received anti-HER2 therapy in this treatment line.

e These patients are registered with a code for “Medical treatment against cancer” with no further specification. This could represent anti-HER2 therapy, chemotherapy, endocrine therapy, or zoledronic acid, so we treat it as missing information on second line therapy.

f Meeting at least one of CLEOPATRA exclusion criteria.

**Statistical methods:** Baseline characteristics were summarized using descriptive statistics and stratified by treatment cohort. The proportion of patients receiving different anti-HER2 therapies (trastuzumab, trastuzumab plus pertuzumab, trastuzumab emtansine, and lapatinib) was reported by year of first-line therapy among all patients initiating anti-HER2 treatment.

Overall survival (OS) from the first anti-HER2 treatment cycle up to eight years was estimated using Kaplan–Meier methods. For comparison, OS curves from the 8-year follow-up of the CLEOPATRA trial [4] were superimposed. Published curves were extracted using WebPlotDigitizer [16] and converted to individual patient data as described by Wei and Royston [17].

To account for potential confounding, adjusted hazard ratios (HRs) with 95% confidence intervals (CIs) were estimated using Cox regression. Covariates included age (continuous), metastatic presentation (de novo vs relapsed), hormone receptor status, income, comorbidity index, polypharmacy, and number of non-cancer hospital contacts (categorized as in Table 1).

In a sensitivity analysis, we fitted a flexible parametric survival model including the same covariates to derive confounding-adjusted survival curves. Regression standardization was applied to obtain marginal survival curves standardized to the covariate distribution of the full cohort. Further explanatory post hoc sensitivity analyses were conducted using Cox regression, estimating adjusted HRs with 95% CIs within subgroups of patients: 1) <75 years, 2) <65 years, 3) de novo metastatic disease, 4) relapsed metastatic disease, 5) hormone receptor–positive disease, and 6) hormone receptor–negative disease.

All statistical analyses were performed in STATA (StataCorp. 2023. Stata Statistical Software: Release 18; College Station, TX), using the stpm3 package [18] to estimate adjusted survival curves and the ipdfc package [17] to derive individual patient data from published Kaplan–Meier curves.

## 3 Results

Of the 329 patients with HER2+ mBC, 78 (24%) were diagnosed in 2012–2014 (pre-CLEOPATRA cohort) and 251 (76%) in 2015–2021 (post-CLEOPATRA cohort). Median age at diagnosis was 54 years; 77 patients (23%) were ≥65 years. Most patients had secondary or higher education (262; 80%) and were in the middle or highest household income quintile (262; 80%). Comorbidity was present in 40 patients (12%), and 190 (58%) had polypharmacy. Overall, 197 patients (60%) were hormone receptor–positive, and 170 (52%) had relapsed from early-stage breast cancer, while 48% presented with de novo metastatic disease. The proportion of relapsed disease differed between cohorts, with a higher proportion in the post-CLEOPATRA cohort (55% vs 40%), which may influence comparisons of survival outcomes between eras (Table 1). Baseline characteristics for patients who did not receive anti-HER2 therapy in both cohorts are provided in Supplemental Table 1.

Previous curative treatment had been administered to 125 patients (38%): 24 (30%) in the pre-CLEOPATRA cohort and 101 (40%) in the post-CLEOPATRA cohort. In the metastatic setting, 151 patients (47%) received second-line anti-HER2 therapy. Among the pre-CLEOPATRA cohort, 19 (59%) received dual anti-HER2 therapy and 6 (19%) trastuzumab emtansine, whereas in the post-CLEOPATRA cohort, 62 (52%) received trastuzumab emtansine and 5 (4%) trastuzumab deruxtecan. Fifty patients (64%) in the pre-CLEOPATRA cohort and 146 (58%) in the post-CLEOPATRA cohort were ineligible for the CLEOPATRA trial. Overall, 196 patients (59%) met at least one CLEOPATRA exclusion criterion (Table 1; details in Supplemental Table 2).

**Fig. 1:**
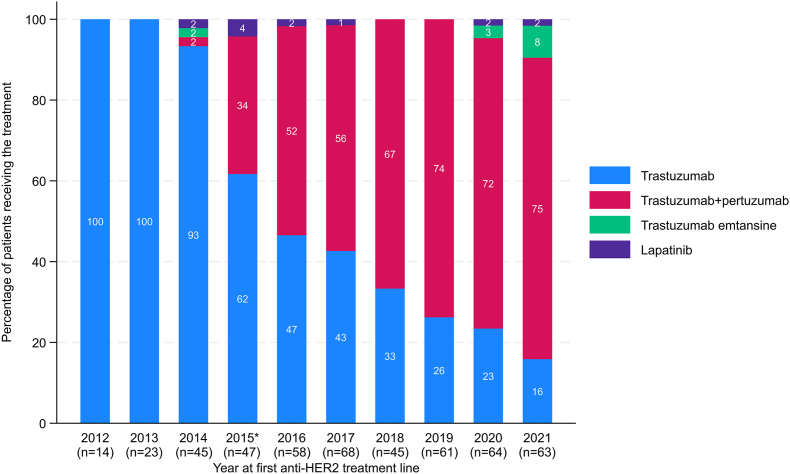
Anti-HER2 therapies used in Norway from 2012 to 2021, according to year of first anti-HER2 treatment line, and percentages of patients receiving the treatment.

Treatment patterns shifted markedly over the study period. In 2012, all patients initiated first-line therapy with trastuzumab monotherapy±chemotherapy, whereas by 2021, 75% received dual therapy with trastuzumab and pertuzumab±chemotherapy. Use of dual blockade increased rapidly following national approval in 2015 and surpassed trastuzumab monotherapy in 2016. The use of trastuzumab emtansine also rose substantially from 2020 to 2021 (3% to 8%) (Fig. 1).

**Fig. 2:**
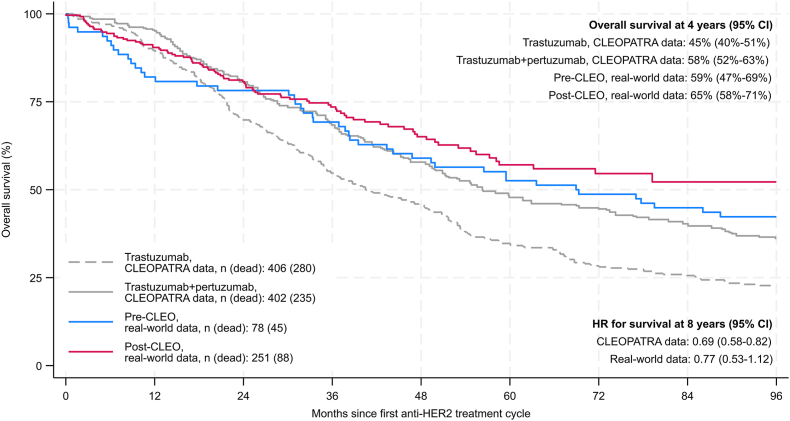
Overall survival (%) according to number of months since first anti-HER2 treatment cycle, for CLEOPATRA trial data (solid grey; dual-anti HER2 therapy, dotted grey: trastuzumab monotherapy) and real-world data (in blue: Pre-CLEOPATRA, in red: Post-CLEOPATRA) and hazard ratios for survival at 8 years with 95% confidence intervals for CLEOPATRA trial data and real-world data.

Overall survival curves demonstrated improved outcomes in the real-world cohorts compared with CLEOPATRA after approximately two years of follow-up. At 48 months, OS was 65% in the post-CLEOPATRA cohort versus 59% in the pre-CLEOPATRA cohort (Fig. 2). Median OS was not reached at 96 months in the post-CLEOPATRA cohort, whereas it was 69 months in the pre-CLEOPATRA cohort. The adjusted hazard ratio for all-cause mortality comparing post-to pre-CLEOPATRA was 0.77 (95% CI, 0.53–1.12) (Fig. 2).

Results from the sensitivity analysis using standardized survival curves were consistent with the main findings (Supplemental Fig. 1). Post hoc sensitivity analyses revealed substantial heterogeneity by metastatic presentation: among patients in the post-CLEOPATRA cohort presenting with de novo metastatic disease, the adjusted HR was 0.53 (95% CI, 0.31–0. 89), whereas among those with relapsed disease, the HR was 1.42 (95% CI, 0.74–2.73) (Supplemental Table 3).

## 4 Discussion

Clinical trials remain essential for establishing treatment efficacy; however, their stringent eligibility criteria frequently exclude older patients and those with comorbidities, limiting the generalizability of trial findings to routine practice. High-quality real-world data are therefore crucial to complement clinical trials and to evaluate treatment effectiveness in broader, less selected populations.

In this nationwide cohort of patients with HER2+ mBC, findings were consistent with the survival benefits demonstrated in the CLEOPATRA trial, observing a trend toward improved outcomes among patients receiving dual anti-HER2 therapy compared with trastuzumab monotherapy. Importantly, nearly 60% of our cohort would not have met CLEOPATRA eligibility criteria, yet survival outcomes with dual anti-HER2 therapy in routine care exceeded those reported in the trial [3,4].

The CLEOPATRA trial [3,4] demonstrated an adjusted hazard ratio (HR) of 0.69 (95% CI, 0.58–0.82) for overall mortality, favoring the addition of pertuzumab. This is comparable to our observed HR of 0.77, although the wider confidence interval in our study (95% CI, 0.53–1.12), likely reflecting the smaller pre-CLEOPATRA cohort (n = 78) compared with the trial population (n = 406), prevents any firm conclusion. In a real-world setting, the survival benefit of adding pertuzumab to trastuzumab appears less pronounced than in the trial. Potential explanations include treatment crossover, whereby patients in the pre-CLEOPATRA cohort later received dual anti-HER2 therapy or other effective anti-HER2 agents, as well as a higher proportion of relapsed disease in the post-CLEOPATRA cohort, which negatively influenced overall survival.

The observed heterogeneity by metastatic presentation may represent a central finding of our study and may help explain the attenuated overall treatment-era comparison. Multiple large HER2-specific studies have shown that patients with recurrent HER2+ mBC experience significantly shorter progression-free and overall survival than those with de novo metastatic disease, with adjusted HRs for death ranging from 0.55 to 0.77 in favor of de novo presentation [19,20]. De novo patients are therapy-naïve and may have lower resistance to systemic therapy and better treatment tolerability. Dual anti-HER2 therapy is considered more toxic than monotherapy, and progressed patients may have lower tolerability for this treatment regimen, resulting in worse survival for these patients. Consistent with this, our sensitivity analyses demonstrated a significant survival advantage for de novo metastatic presentation in the post-CLEOPATRA cohort (adjusted HR 0.53, 95% CI 0.31–0.89; Supplemental Table 3).

Several real-world studies report comparable survival among patients treated with dual-anti HER2 therapies as demonstrated in CLEOPATRA [7,9]. However, comparisons across treatment eras reflect not only the introduction of dual HER2 blockade but also concurrent changes in subsequent therapies, diagnostics, and supportive care, making it difficult to isolate the independent contribution of first-line treatment. We observed inferior survival outcomes during the first year of treatment compared to CLEOPATRA, likely reflecting the inclusion of patients with reduced life expectancy due to brain metastasis or compromised baseline performance status. After the second year of follow-up, patients had better survival than in CLEOPATRA, which is likely multifactorial. First, the therapeutic landscape has evolved substantially since CLEOPATRA enrollment (2008–2010). The introduction of effective subsequent-line therapies, primarily trastuzumab emtansine (EMILIA) [19] and trastuzumab deruxtecan (DESTINY-Breast03) [20] has markedly improved survival following disease progression and likely extended overall survival from the time of first-line therapy. This is in line with findings from the French ESME cohort [9], including close to 4000 HER2+ mBC patients from 2008 to 2016, demonstrating a marked, time-dependent median OS improvement from 39 months in 2008 to 58 months in 2013 and not reached from 2014 and onwards, attributed to the implementation of new anti-HER2 treatments in clinical practice. Second, advances in local management of intracranial disease may further contribute to prolonged survival [21], such as the use of stereotactic radiotherapy and surgical treatment of brain metastasis. We observed a trend towards improved prognosis for HER2+ patients with brain metastasis in our data, but the sample was unfortunately too small for conclusions to be drawn. Third, the Norwegian public health-care system ensures free and universal access to anti-HER2 therapy and follow-up, reducing disparities in treatment availability compared with the more heterogeneous health systems represented in CLEOPATRA. Finally, although our study is population-based, patients who receive systemic therapy in clinical practice often represent a selected, fitter subgroup, and the exclusion of patients who did not receive recommended first line therapy means that our findings do not represent all patients with HER2-positive metastatic breast cancer at the population level. Patients who did not receive anti-HER2 therapy during the study period (as presented in Supplemental Table 1), were older and more comorbid than treated patients. Collectively, these factors provide plausible explanations for the superior survival observed in our real-world setting compared with the pivotal trial.

## 5 Strengths and limitations

The major strength of this study is its population-based design minimizing the risk of selection bias. Further, there is equitable access and near-complete capture of therapy data, and longitudinal follow-up of patients which allowed for clear pre- and post-CLEOPATRA cohorts illustrating the impact of practice change at a national level. The linkage of several validated national registries provided reliable information on tumor characteristics, systemic therapies, prescriptions and mortality. The high degree of internal validity enhances generalizability to countries with a similar health system.

Limitations include the lack of clinical detail such as performance status, metastatic burden and metastatic sites, which restrict adjustment for prognostic factors used in CLEOPATRA. Although the study is population-based, frailer patients who are not offered therapy are not included, which may overestimate survival in both cohorts relative to a truly unselected population. A very small number of patients may have received pertuzumab or T-DM1 privately before national approval; these data are not recorded, though the impact is likely negligible due to the limited use of private health care in Norway [22]. Improvements in supportive care, diagnostics and availability of subsequent line therapies contribute to survival gains. This makes it difficult to isolate the effect of first-line CLEOPATRA therapy alone.

## 6 Conclusion

In this nationwide cohort, patients with HER2+ metastatic breast cancer treated with first-line dual anti-HER2 therapy experienced durable and clinically meaningful long-term survival in routine clinical practice. Survival estimates were broadly consistent with those reported in the CLEOPATRA trial, although direct equivalence cannot be inferred given the observational design and the evolving treatment landscape. The observed survival differences between treatment eras likely reflect both the introduction of dual HER2 blockade and concurrent advances in subsequent-line therapies, diagnostics, and supportive care. Notably, substantial heterogeneity by metastatic presentation was observed, with more pronounced benefit among patients with de novo metastatic disease, suggesting that treatment effects may differ by timing of metastatic disease presentation. Taken together, these findings are consistent with the effectiveness of dual HER2 blockade observed in clinical trials and illustrate how comprehensive, population-based registries can contribute to the interpretation of trial findings in routine clinical practice.Future research should prioritize evaluating outcomes in older and comorbid patient populations, who continue to be underrepresented in clinical trials.

## Ethics statement

This research was performed in compliance with relevant laws and institutional guidelines and has been approved by the Regional Committees for Medical and Health Research Ethics, Southeast Norway (nr 2018/775).

## Data statement

All data are available in the Cancer Registry of Norway. Further information is available from the corresponding author upon request.

## CRediT authorship contribution statement

**Kathrine F. Vandraas:** Conceptualization, Formal analysis, Resources, Visualization, Writing – original draft, Writing – review & editing. **Sarah Hjorth:** Conceptualization, Data curation, Formal analysis, Methodology, Visualization, Writing – review & editing. **Cassia B. Trewin-Nybråten:** Conceptualization, Formal analysis, Methodology, Visualization, Writing – review & editing. **Bettina K. Andreassen:** Conceptualization, Formal analysis, Visualization, Writing – review & editing. **Egil S. Blix:** Writing – review & editing. **Bjørn Naume:** Writing – review & editing. **Edoardo Botteri:** Conceptualization, Formal analysis, Visualization, Writing – review & editing. **Giske Ursin:** Writing – review & editing. **Nathalie C. Støer:** Conceptualization, Formal analysis, Funding acquisition, Project administration, Supervision, Visualization, Writing – review & editing.

## Declaration of competing interest

The authors declare the following financial interests/personal relationships which may be considered as potential competing interests:

Egil S. Blix reports a relationship with AstraZeneca that includes: consulting or advisory. Egil S.Blix reports a relationship with Daiichi Sankyo Inc that includes: consulting or advisory. Egil S. Blix reports a relationship with Eli Lilly that includes: consulting or advisory. Egil S. Blix reports a relationship with Novartis that includes: consulting or advisory. Egil S. Blix reports a relationship with Pfizer that includes: consulting or advisory. Egil S. Blix reports a relationship with Roche that includes: consulting or advisory. If there are other authors, they declare that they have no known competing financial interests or personal relationships that could have appeared to influence the work reported in this paper.

## Appendix A Supplementary data

Multimedia component 3

Fig. S1 Flowchart of study population.

Fig. S2 Standardized survival (%) according to number of months since first anti-HER2 treatment cycle for real-world data (in blue: Pre-CLEOPATRA, in red: Post-CLEOPATRA) and for CLEOPATRA trial data (solid grey; dual-anti HER2 therapy, dotted grey: trastuzumab monotherapy), and hazard ratios for survival at 8 years with 95% confidence intervals for CLEOPATRA trial data and real-world data.
