# Supplementary material for: Translating CLEOPATRA into routine practice: National treatment patterns and survival for patients with HER2-positive metastatic breast cancer
Source: Breast. 2026 Jun 27;89:104852. doi: 10.1016/j.breast.2026.104852 (PMC13355192; doi:10.1016/j.breast.2026.104852)
Supplement: Multimedia component 3 [file mmc3.docx]

|  | n | Median follow-up per patient (months) | n deaths | Crude HR (95% CI) | Adjusted^a^ HR (95% CI) |
| --- | --- | --- | --- | --- | --- |
| **Main analysis** | | | | | |
| Pre-CLEOPATRA | 78 | 69.1 | 45 | Reference | Reference |
| Post-CLEOPATRA | 251 | 35.9 | 88 | 0.81 (0.56 -1.17) | 0.77 (0.53-1.12) |
| ***Post hoc* subgroup analyses** | | | | | |
| Restricted to patients aged <75 years | | | | | |
| Pre-CLEOPATRA | 71 | 77.7 | 39 | Reference | Reference |
| Post-CLEOPATRA | 234 | 36.5 | 81 | 0.86 (0.58-1.28) | 0.81 (0.54-1.22) |
| Restricted to patients aged <65 years | | | | | |
| Pre-CLEOPATRA | 62 | 92.2 | 31 | Reference | Reference |
| Post-CLEOPATRA | 190 | 39.8 | 60 | 0.89 (0.57-1.39) | 0.83 (0.52-1.32) |
| Restricted to patients with *de novo* metastatic disease | | | | | |
| Pre-CLEOPATRA | 47 | 49.9 | 32 | Reference | Reference |
| Post-CLEOPATRA | 112 | 41.3 | 32 | 0.50 (0.30-0.82) | 0.53 (0.31-0.89) |
| Restricted to patients with relapsed metastatic disease |  |  |  |  |  |
| Pre-CLEOPATRA | 31 | 96.0 | 13 | Reference | Reference |
| Post-CLEOPATRA | 139 | 33.8 | 56 | 1.42 (0.76-2.65) | 1.42 (0.74-2.73) |
| Restricted to patients with hormone receptor positive disease | | | | | |
| Pre-CLEOPATRA | 50 | 66.4 | 29 | Reference | Reference |
| Post-CLEOPATRA | 147 | 37.6 | 47 | 0.74 (0.46-1.19) | 0.71 (0.43-1.15) |
| Restricted to patients with hormone receptor negative disease | | | | | |
| Pre-CLEOPATRA | 28 | 73.3 | 16 | Reference | Reference |
| Post-CLEOPATRA | 104 | 33.9 | 41 | 0.90 (0.50-1.63) | 0.89 (0.48-1.63) |

^a^The hazard ratio was adjusted for age, income, *de novo*/relapsed disease, hormone receptor status, comorbidity, polypharmacy, and number of hospital contacts in the year before inclusion. In the analysis stratified by *de novo/*relapsed metastatic disease, the variable *de novo*/relapsed disease was not included in the adjustment model. In the analyses stratified by hormone receptor status, the variable hormone receptor status was included in the adjustment model.

HR: hazard ratio. CI: Confidence interval.
