# Supplementary material for: Translating CLEOPATRA into routine practice: National treatment patterns and survival for patients with HER2-positive metastatic breast cancer
Source: Breast. 2026 Jun 27;89:104852. doi: 10.1016/j.breast.2026.104852 (PMC13355192; doi:10.1016/j.breast.2026.104852)
Supplement: Fig. S1 — Flowchart of study population. [file mmc4.docx]

4,692 patients with HER2+ carcinoma of the breast from 2012-2021

3,928 patients diagnosed with stage I-III HER2+ carcinoma who did not experience distant recurrence before 31 December 2021

764 patients with metastatic HER2+ carcinoma

*Men: 0

*Previous cancer according to CRN: 88

*Assumed previous cancer from treatment information: <5

*Other cancer at date of first breast cancer diagnosis: <5

*Diagnosed at autopsy, dead or censored at the date of metastatic diagnosis: <5

*Missing data on adjustment variables: 8

*Not registered with trastuzumab monotherapy (2012-2014) or trastuzumab+pertuzumab (2015-2021): 348

329 patients included in the study
