# Supplementary figures and images for: Translating CLEOPATRA into routine practice: National treatment patterns and survival for patients with HER2-positive metastatic breast cancer
Source: Breast. 2026 Jun 27;89:104852. doi: 10.1016/j.breast.2026.104852 (PMC13355192; doi:10.1016/j.breast.2026.104852)

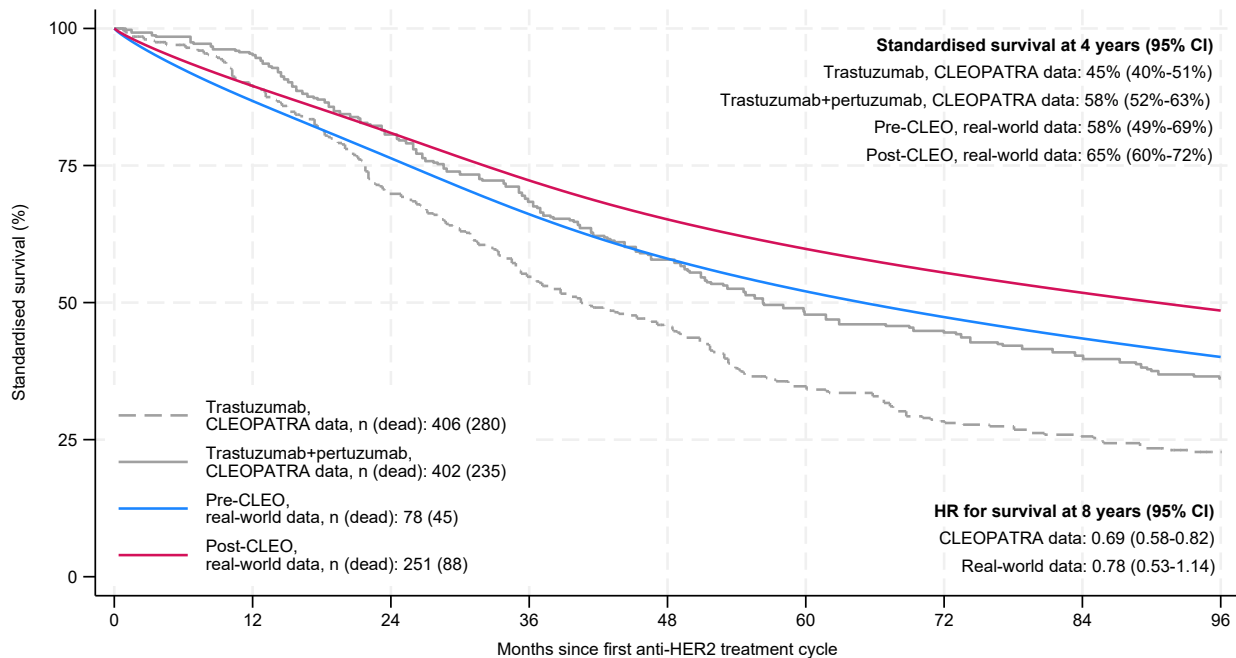

Supplement: Fig. S2 — Standardized survival (%) according to number of months since first anti-HER2 treatment cycle for real-world data (in blue: Pre-CLEOPATRA, in red: Post-CLEOPATRA) and for CLEOPATRA trial data (solid grey; dual-anti HER2 therapy, dotted grey: trastuzumab monotherapy), and hazard ratios for survival at 8 years with 95% confidence intervals for CLEOPATRA trial data and real-world data. [file mmc5.pdf]
